# Aluminum induces cross-resistance of potato to *Phytophthora infestans*

**DOI:** 10.1007/s00425-013-2008-8

**Published:** 2013-12-18

**Authors:** Magdalena Arasimowicz-Jelonek, Jolanta Floryszak-Wieczorek, Kinga Drzewiecka, Jagna Chmielowska-Bąk, Dariusz Abramowski, Karolina Izbiańska

**Affiliations:** 1 Department of Plant Ecophysiology, Faculty of Biology, Adam Mickiewicz University, Umultowska 89, 61-614 Poznan, Poland; 2 Department of Plant Physiology, Poznan University of Life Sciences, Wołynska 35, 60-637 Poznan, Poland; 3 Chemistry Department, Poznan University of Life Sciences, Wojska Polskiego 28, 60-637 Poznan, Poland

**Keywords:** Aluminum, Biotic stress, Cross-resistance, Nitric oxide, *S*-Nitrosothiols, Salicylic acid

## Abstract

The phenomenon of cross-resistance allows plants to acquire resistance to a broad range of stresses after previous exposure to one specific factor. Although this stress–response relationship has been known for decades, the sequence of events that underpin cross-resistance remains unknown. Our experiments revealed that susceptible potato (*Solanum tuberosum* L. cv. Bintje) undergoing aluminum (Al) stress at the root level showed enhanced defense responses correlated with reduced disease symptoms after leaf inoculation with *Phytophthora infestans*. The protection capacity of Al to subsequent stress was associated with the local accumulation of H_2_O_2_ in roots and systemic activation of salicylic acid (SA) and nitric oxide (NO) dependent pathways. The most crucial Al-mediated changes involved coding of NO message in an enhanced *S*-nitrosothiol formation in leaves tuned with an abundant SNOs accumulation in the main vein of leaves. Al-induced distal NO generation was correlated with the overexpression of PR-2 and PR-3 at both mRNA and protein activity levels. In turn, after contact with a pathogen we observed early up-regulation of SA-mediated defense genes, e.g. *PR1*, *PR*-*2*, *PR*-*3* and *PAL*, and subsequent disease limitation. Taken together Al exposure induced distal changes in the biochemical stress imprint, facilitating more effective responses to a subsequent pathogen attack.

**Electronic supplementary material:**

The online version of this article (doi:10.1007/s00425-013-2008-8) contains supplementary material, which is available to authorized users.

## Introduction

Acid soils account for approximately 35 % of arable land and are one of the most important limitations to plant production worldwide (Ryan et al. 2011). The restriction of crop growth and yield in soils with pH below 5.0 is mainly due to the formation and migration of phytotoxic aluminum ions Al^3+^. The primary target of phytotoxic Al^3+^ are plant roots, in which inhibition of root elongation and alternation of root architecture is observed even within minutes of the exposure (Doncheva et al. 2005). It is well documented that Al can disrupt many crucial physiological processes at both cellular and molecular levels, including nutrient and water uptake, intracellular transport, cell division and elongation, cell signaling and redox homeostasis (Barceló and Poschenrieder 2002).

Aluminum toxicity is not the only cause of crop production limitation. Plants are continuously exposed to a challenge by a broad range of stresses. In the course of evolutionary changes plants have developed mechanisms facilitating avoidance or active control of adverse environmental conditions. Effective plant defense responses are usually realized by the activation of specific signaling components with the expression of a battery of target genes, leading to tolerance or resistance mechanisms. An increasing body of evidence shows that plant exposure to one specific stress leads to acquired resistance to another stress. This phenomenon is known as cross-tolerance, cross-resistance or multiple-stress resistance and was shown for different types of stress (Steinberg 2012). For example, ozone exposure can induce resistance to virulent phytopathogenic *Pseudomonas syringae* strains in *Arabidopsis* and to tobacco mosaic virus in tobacco plants (Yalpani et al. 1994; Sharma et al. 1996). In barley, salt-induced osmotic stress was correlated with resistance to powdery mildew (Wiese et al. 2004), while in tomato drought stress enhanced resistance to *Botrytis cinerea* (Achuo et al. 2006). In relation to abiotic stresses it was demonstrated that wounding increased salt stress tolerance in tomato (Capiati et al. 2006), whereas cold acclimation can increase heat tolerance of winter rye, UV radiation enhanced drought tolerance in *Pisum sativum* and *Triticum aestivum* (Alexieva et al. 2001), heat shock improved tolerance of maize to heat, chilling, salt and drought (Gong et al. 2001).

Accumulating data strongly support that plant responses to both biotic and abiotic stresses are mediated by common signals, i.e. reactive oxygen and nitrogen species (ROS and RNS), calcium, salicylic acid (SA), jasmonic acid (JA) and abscisic acid (ABA) (Tippmann et al. 2006). The orchestration of these signals might be a central component controlling cross-tolerance, at least at the cellular level (Bowler and Fluhr 2000). On the other hand, there is evidence that the cross-tolerance phenomenon could be governed by an overriding signal molecule. For example, Asselbergh et al. (2008) suggested that ABA is involved in controlling a global shift between plant responses to abiotic and biotic stresses. In turn, strictly controlled generation of hydrogen peroxide (H_2_O_2_) in transgenic tobacco (Cat1AS) that has a decreased ROS scavenging capacity resulted in both local and systemic induction of a pathogenesis-related protein and subsequently in inducible local resistance to bacterial pathogens (Chamnongpol et al. 1998). According to Bowler and Fluhr (2000), the cross-tolerance phenomenon occurs when the whole plant is exposed to the primary experience, or when systemic signals are also stimulated to ensure robust systemic resistance phenotypes. The long-distance modified signaling can lead to systemic acquired resistance (SAR) to a wide spectrum of pathogens and in relation to improved responses to abiotic stresses it is connected with systemic acquired acclimation (SAA). Interestingly, it has been reported that SAA involves a novel signal or a complex interaction between known signals distinct from pathogen-stimulated systemic acquired resistance, since SAA was transmitted in *Arabidopsis* mutants that perturb ABA, JA, SA, and SAR signaling (Rossel et al. 2007).

Although the importance of redox signals in networks that underpin cross-tolerance is strongly emphasized (Pastori and Foyer 2002), the role of nitric oxide (NO) in this stress response relationship remains puzzling. However, the evidence that NO is also involved in signaling defense during a plant–pathogen interaction and/or tolerance response to abiotic stress factors has been well documented in numerous experiments (for review see e.g.: Moreau et al. 2010).

In this study, we showed that aluminum exposure triggered distal signaling in potato plants, facilitating resistance to a subsequent attack by the oomycete pathogen *Phytophthora infestans.* Moreover, we provided evidence that the sequence of events that link perception and transduction of primary Al-stress to biotic defense responses involve distal conversion of the NO message. To this end we monitored the metabolic status of NO at root-stem-leaves levels, including NO orchestration with H_2_O_2_ and SA after exposure to moderate aluminum stress enabling establishment of protection responses to the oomycete pathogen.

## Materials and methods

### Plant growth conditions and stress treatment

Sterile potato plants (*Solanum tuberosum* L.) of cv. Bintje, susceptible to *Phytophthora infestans*, derived from in vitro tissue culture (Plant Breeding and Acclimatization Institute, Research Division at Bonin, Poland) were grown hydroponically in plastic containers of 4 dm^3^ in capacity. A modified Hoagland solution was used. After 3 weeks potato plants were transferred to a nutrient solution at pH 4.3 either supplemented with AlCl_3_ to a final concentration of 250 μM (Al stress), or without AlCl_3_ (acidic control) for 48 h. During the experiment the medium was aerated and after each week it was exchanged. The experiment was carried out in a phytochamber with 16 h of light (180 μmol m^−2^ s^−1^) at 18 ± 2 °C and 60 % humidity.

### Pathogen culture

*Phytophthora infestans* race 1.3.4.7.10.11 (MP 946) as a virulent isolate of the pathogen was kindly supplied by the Plant Breeding and Acclimatization Institute (IHAR), Research Division at Młochów, Poland. *Phytophthora infestans* was grown on a cereal-potato medium with an addition of dextrose. A zoospore suspension of *P. infestans* was prepared exactly as described by Floryszak-Wieczorek (2000).

### Plant challenge inoculation with *P. infestans*

Each potato plant was challenge-inoculated by spraying leaves with 5 ml of a *P. infestans* zoospore suspension at a zoospore concentration of 2.0 × 10^5^ per ml of water. For disease assessment inoculated plants were first kept for 12 h at 100 % humidity in the dark at 18 °C. Next plants were moved to a growth chamber under controlled conditions.

### Assessment of disease index

The index of disease development on potato leaves at 24, 48, 72 h after *P. infestans* inoculation (hpi) represented the percentage of leaf area covered by late blight symptoms. Values are means of the average disease index from leaves of Al-treated and then challenge-inoculated plants from three independent experiments.

### NO detection by CLSM

Nitric oxide formation was detected using a fluorescent dye Cu-FL according to Lim et al. (2006). Copper-complex of FL (2-{2-chloro-6-hydroxy-5-[2-methylquinolin-8-ylamino)methyl]-3-oxo-3H-xanthen-9-l}benzoic acid) was prepared as 1 mM water stock solution according to manufacturer’s instructions (Strem Chemicals, Newburyport, MA, USA). Leaf and root sections were incubated for 30 min at room temperature with Cu-FL at a final concentration of 10 μM in 10 mM Tris–HCl buffer, pH 7.2. The incubation solution was then removed and sections were studied under an epifluorescence microscope (Axiostar plus, Zeiss) equipped with an AxioCam MRc 5 camera (Zeiss). For observation of NO-FL fluorescence the Filter Set 16 (Zeiss) (ex. 485/20; em. 515 nm) were used. Fluorescence intensity in extracts was determined at Fluorescence Spectrometer Perkin Elmer LS 50B (UK) using 488 and 516 nm for excitation and emission, respectively.

### Measurement of SA

Salicylic acid in free form (SA), as well as that conjugated as a glucoside (SAG), were determined according to the methodology recommended by Yalpani et al. (1993). Plant tissues were ground in liquid nitrogen to a fine powder from which approximately 0.5 g was taken for analysis. Salicylic acid was extracted twice with methanol. After centrifugation, the supernatant was divided into two equal parts and the solvent was evaporated to dryness under a stream of nitrogen. A 5 % solution of trichloroacetic acid was added to one part and then SA was extracted three times into ethyl acetate:cyclopentane:isopropanol (100:99:1, by vol.). To determine the total (free and glucoside bound) salicylic acid (TSA), 40 units of β-glucosidase (Sigma) in acetate buffer (0.1 M, pH 5.2) were added to the second part of the dry extract and incubated for 90 min at 37 °C. The reaction was terminated by the addition of 5 % trichloroacetic acid and then salicylic acid was extracted as described above. After solvent evaporation, the dry residue was dissolved in a mobile phase (0.2 M acetate buffer, pH 5.0; 0.5 mM EDTA) and analyzed by the HPLC method coupled with fluorometric detection with a Waters chromatograph composed of 2,699 Separation Module Alliance and 2,475 Multi-λ Fluorescence Detector (Waters Corp., Milford, MA, USA). Chromatographic separation was performed on a Spherisorb ODS2 Waters column (3 μm, 4.6 × 10 mm) (Waters Corp., Wexford, Ireland). Detection parameters were as follows: 295 nm for excitation and 405 nm for emission. The content of the salicylic acid released from its glucoside was calculated as the difference between assays without and with glucoside enzymatic degradation (SAG = TSA − SA). The relationship between the peak area and the concentration of salicylic acid was found to be highly linear with a regression coefficient *R* ^2^ > 0.9999. The recovery of the standard added to samples amounted to 89 and 86 % for SA and TSA assays, respectively.

### Measurement of H_2_O_2_

H_2_O_2_ concentration was assayed spectrophotometrically using the titanium (Ti^4+^) method described by Becana et al. (1986). Leaves, stems and roots (0.25 g) were homogenized in 3 ml of 0.1 M potassium phosphate buffer (pH 7.8). After centrifugation (15,000*g* for 30 min), the supernatant was used for further assays. The reaction mixture (1.5 ml) contained 0.1 M potassium phosphate buffer (pH 7.8), enzymatic extract (400 μl) and titanium reagent. Titanium reagent was prepared on the day of assay by mixing 0.6 mM solution of 4-(2-pyridylazo) resorcinol and 0.6 mM potassium titanium tartrate at a 1:1 ratio. The prepared solution was kept in an ice bath. The concentration of H_2_O_2_ was estimated by measuring absorbance at a wavelength of 508 nm against a calibration curve and expressed as mmol H_2_O_2_ per 1 g fresh weight (FW).

### GSNOR activity (EC 1.2.1.46)

The GSNOR activity was determined according to the procedure proposed by Barroso et al. (2006) with minor modifications. All operations were carried out at 0–4 °C. Fresh leaves, stems and roots (0.5 g) were homogenized in 0.1 M Tris–HCl buffer, pH 7.5 (1:4 w/v) containing 0.2 % Triton X-100 (v/v), 10 % glycerol (v/v), 0.1 mM EDTA, 2 mM DTT and centrifuged at 27,000*g* for 25 min. Supernatants were passed through Sephadex G-25 gel filtration columns (Illustra NAP-10 from GE Healthcare), then immediately through Amicon Ultra 3K Filters (Millipore) and served as the enzyme extract. The 1 ml volume of the assay reaction mixture contained 0.5 mM EDTA, 0.2 mM NADH, 0.4 mM GSNO and 30 μl enzyme extract in 25 mM Tris–HCl buffer, pH 8.0. The reaction was run at 25 °C and initiated with an addition of GSNO (Sigma Aldrich). NADH oxidation was determined at 340 nm and rates of NADH consumed at min^−1^ were calculated using an extinction coefficient of 6,220 M^−1^ cm^−1^.

### β-1,3-Glucanase activity (EC 3.2.1.6)

The β-1,3-glucanse activity was determined according to the procedure proposed by Abeles and Forrence (1970) in a colorimetric assay utilizing laminarin as a substrate. All operations were carried out at 0–4 °C. Fresh leaves and roots (0.25 g) were homogenized in 0.05 M potassium-acetate buffer, pH 5.0 (1:16; w/v), containing 0.125 g Polyclar AT and then centrifuged at 15,000*g* for 25 min. Supernatants (0.5 ml) were added to 0.5 ml 2 % (w/v) laminarin aqueous solution and incubated for 2 h at 50 °C. The reaction was stopped by adding 3 ml of the dinitrosalicylic reagent and probes were heated for 5 min at 100 °C. The probes were then cooled to 25 °C, the contents were diluted with water to 1:10 and the optical density was read at 500 nm. The β-1,3-glucanase activity was determined as a level of reducing sugars produced and served as glucose equivalents in μmol glucose mg^−1^ protein min^−1^.

### Chitinase activity (EC 3.2.1.14)

Chitinase activity was determined according to Derckel et al. (1996) with some modifications. Fresh leaves and roots (0.25 g) were homogenized in 50 mM sodium buffer, pH 5.0 (1:10; w/v) containing 0.125 g Polyclar AT and then centrifuged at 15,000*g* for 25 min. The diluted (1:10) enzyme extract (0.2 ml) was incubated at 25 °C in an Eppendorf microcentrifuge tube with 0.2 ml of CM-Chitin-RBV (Loewe Biochemica, Germany) and 0.4 ml of 50 mM sodium acetate buffer pH 5.0 for 60 min. Next the reaction was stopped by the addition of 0.2 ml 2 M HCl and cooled on ice for 10 min. After the centrifugation at 10,000*g* for 5 min, the mixture was transferred to a plastic cuvette and absorbance was measured at 550 nm. Chitinase activity was expressed as units per mg protein, with one unit equal to 1 μg CM-chitin-RBV hydrolyzed per minute (μg CM-chitin-RBV/min).

### Quantification of total SNOs

Total SNO content was determined by chemiluminescence using a Sievers^®^ Nitric Oxide Analyzer NOA 280i (GE Analytical Instruments, Boulder, CO, USA) according to the procedure proposed by Valderrama et al. (2007) with minor modifications. The detection of SNOs depends on the reductive decomposition of nitroso compounds by an iodine/triiodide mixture in the presence of copper. Gaseous NO released from the mercury-induced decomposition of SNOs is measured by gas-phase chemiluminescence at the PMT device. Fresh leaves, stems and roots (0.5 g) were homogenized in Tris–HCl 0.1 M buffer pH 7.5 (1:4, w/v) containing 100 μM DTPA, 1 mM EDTA, 1 mM EGTA, 1 mM PMSF, 0.1 mM neocuproine, 3.5 % (w/v) PVPP, 0.25 % (v/v) Triton X-100 and centrifuged at 3,000*g* for 10 min. The supernatants were incubated with 10 mM NEM (*N*-ethylmaleimide) for 15 min at 4 °C and subsequently two aliquots were prepared for each sample. To remove nitrite one aliquot was incubated for 15 min with 10 mM sulphanilamide at 4 °C. To eliminate nitrite and decompose SNOs the next aliquot was treated with 10 mM sulphanilamide and 7.3 mM HgCl_2_ for 15 min at 4 °C. The difference between detected signals obtained from these aliquots demonstrated the total SNO content. Due to SNO sensitivity to light the whole procedure was performed under a red safety light.

### Immunohistochemical studies of SNOs

Detection of SNOs by fluorescence microscopy using the fluorescent Alexa Fluor 405 Hg-Link reagent phenylmercury was performed according to Corpas et al. (2008) with some modifications. Potato root and leaf segments were incubated at 25 °C for 1 h in darkness with 10 mM NEM prepared in ethanol, and then were washed three times in 10 mM Tris–HCl buffer, pH 7.4, for 15 min each. Then they were incubated in the dark with 10 μM Alexa Fluor 405 Hg-link phenylmercury (Molecular Probes, USA) for 1 h at 25 °C. After being washed three times in 10 mM Tris–HCl buffer the sections were analyzed with an epifluorescence microscope (Axiostar plus, Zeiss) equipped with an AxioCam MRc 5 camera (Zeiss) using standard filters for Alexa Fluor 405 blue fluorescence (excitation 401 nm; emission 421 nm). For background staining control sections were incubated with β-mercaptoethanol plus Alexa Fluor 405 and without NEM.

### Gene expression measurement

The RNA was isolated from 200 mg of frozen leaf and root tissues using TriReagent (Sigma). The obtained RNA was purified with the use of a Deoxyribonuclease Kit (Sigma). For the reverse transcription 1 μl of RNA from every experimental variant was processed with a Reverse Transcription Kit (Thermo Scientific Fermentas) according to the manufacturer’s instructions. Real-time PCR was performed on a RotorGene 6000 Thermocycler. The reaction mixture contained 0.1 μM of each primer, 1 μl of 5× diluted cDNA, 10 μl of the Power SYBR Green PCR Master mix (Applied Biosystems) and DEPC treated water to the total volume of 20 μl. The real-time PCR reaction conditions included an initial 5-min denaturation at 95 °C, followed by 55 cycles consisting of 10 s at 95 °C, 20 s at 55 °C and 30 s at 72 °C. The reaction was finalized by denaturation at a temperature rising from 72 to 95 °C by one degree at every 5 s. Reaction specificity was confirmed by the occurrence of one peak in the melting curve analysis. The data were normalized to two reference genes encoding elongation factor (ef1α, AB061263) and 18S RNA (X67238). All used primers are presented in Suppl. Table S1. The Ct values were determined with the use of a Real-time PCR Miner (Zhao and Fernald 2005) and the relative gene expression was calculated with the use of efficiency corrected calculation models presented in Pfaffl (2004).

### Protein concentration

Protein concentration was assayed according to Bradford (1976) using bovine serum albumin as a standard.

### Statistical analysis

All experiments included three independent experiments carried out in at least three technical replications. For each experiment means of the obtained values were calculated along with standard deviations. The analysis of variance was performed and the least significant differences (LSDs) between means were determined using Tukey’s test at the level of significance *α* = 0.05.

## Results

### Aluminum exposure activated key markers of plant defense responses in both roots and leaves

**Fig. 1:**
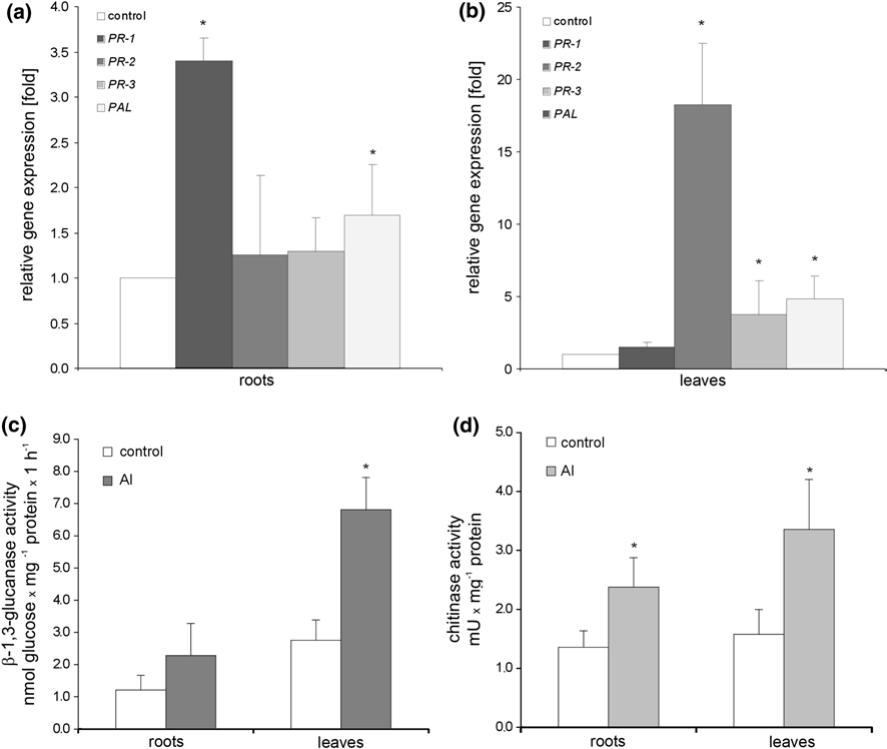
The qRT-PCR analysis of *PR*-*1*, *PR*-*2*, *PR*-*3* and *PAL* gene expression in roots (**a**) and leaves (**b**) of potato exposed to 250 μM AlCl3 at 48 h. β-1,3-Glucanase (**c**) and chitinase (**d**) activities in roots and leaves of Al-treated potato. *Asterisks* indicate values that differ significantly from the non-treated (control) potato plants at *P* < 0.05, *n* = 3

Potato exposure to Al significantly up-regulated the expression of two of the four studied defense-related genes in roots including *PR*-*1* and *PAL*. However, the highest expression level (over threefold increase) was noted for *PR*-*1* (Fig. 1a). In leaves mRNA coding for *PR*-*2*, *PR*-*3* and *PAL* was effectively increased, ranging from 3- to 18-fold increase for *PR*-*3* and *PR*-*2*, respectively (Fig. 1b). A distinct pattern of expression was observed for *PR*-*1*, since Al did not elicit the significant rise of PR-1 transcript accumulation in distal organs. Furthermore, Al-mediated up-regulation of *PR*-*2* and *PR*-*3* in leaves was correlated in time with an elevated β-1,3-glucanse and chitinase activity (Fig. 1c, d).

It worth pointing that the used Al concentration (250 μM AlCl_3_) includes in the tolerance limit noted for potato plants, since inhibition of root growth by 50 %, known as the tolerance index, was observed at Al concentration of 500 μM (see Supporting Information Fig. S1).

### Aluminum triggered distal signaling in potato plants

**Fig. 2:**
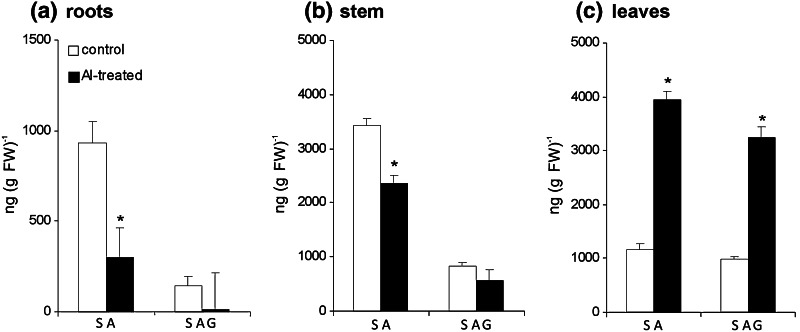
The level of free SA and SA conjugated with Glc in potato plants exposed to 250 μM AlCl3 at 48 h. SA and SAG content was measured in roots (**a**), shoots (**b**) and leaves (**c**). *Asterisks* indicate values that differ significantly from the non-treated control plants at *P* < 0.05

To determine if root exposure to Al may modulate a post-stress signaling network in the upper organs, the levels of SA, NO and H_2_O_2_ in stems and leaves were also determined. The relatively short-term exposure of potato plants to Al provoked a marked, by ca. 70 %, decrease in free SA content in roots (Fig. 2a). The abated level of SA accumulation in response to Al treatment was observed in potato stems as well; however, the steady-state level of free SA was higher than in roots (Fig. 2b). Surprisingly, the level of free SA in leaves increased by over threefold in response to Al treatment (Fig. 2c).

The production of salicylic acid beta-glucoside (SAG) was also reduced in response to Al treatment in both roots and stems (Fig. 2a, b). In turn, the content of the storage form of the analyzed signal in leaves was over three times higher in comparison to non-treated plants (Fig. 2c).

**Fig. 3:**
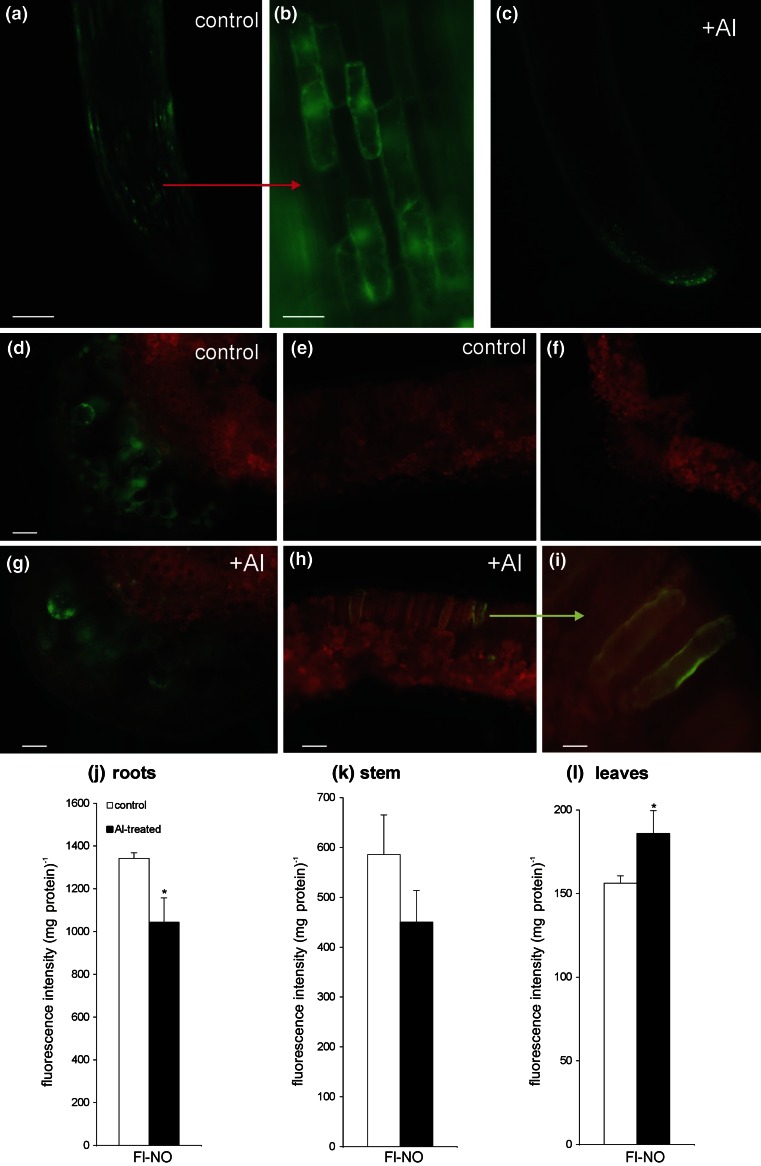
Bio-imaging of NO with a Cu-FL fluorescent probe in potato roots (**a**–**c**) and leaves (**d**, **e**, **g**–**i**) at 48 h after root exposure to 250 μM AlCl_3_. Images show general phenomena representative of three individual experiments; control of background where the fluorescent probe was omitted (**f**). *Bars* indicate 200 μm (**d**–**f**), 100 μm (**a**, **c**, **g**, **h**) and 20 μm (**b**, **i**). Measurement of FL-NO fluorescence in extracts of potato roots (**j**), stems (**k**) and leaves (**l**) exposed to aluminum. NO production was assayed spectrofluorimetrically using a selective NO sensor (Cu-FL). FL-NO fluorescence intensity represents mean values for the average of data ± SD of three independent experiments

The localization of NO was analyzed by fluorescence microscopy using the selective fluorescent probe cupper-complex (Cu-FL), where green fluorescence corresponds to the location of NO. Based on FL-NO fluorescence we observed that the control potato generated considerable amounts of NO only in the root apical zone (Fig. 3a, b). The treatment with 250 μM AlCl_3_ for 48 h significantly diminished NO synthesis in the elongation and differentiation zone of potato roots (Fig. 3c). In addition, quantitative measurement of FL-NO fluorescence in potato extracts confirmed a decrease in NO synthesis in roots and revealed diminished the signal generation in stems as well (Fig. 3j, k). In leaves NO-dependent fluorescence was increased (Fig. 3g–i, l). The enhanced NO formation was found particularly in the parenchyma and in single cells of palisade mesophyll (Fig. 3g–i). The application of 1 mM PTIO almost completely eliminated green fluorescence in Al-treated potato (see Supporting Information Fig. S2). The potato leaf cross section was presented in Supporting Information (Fig. S3).

**Fig. 4:**
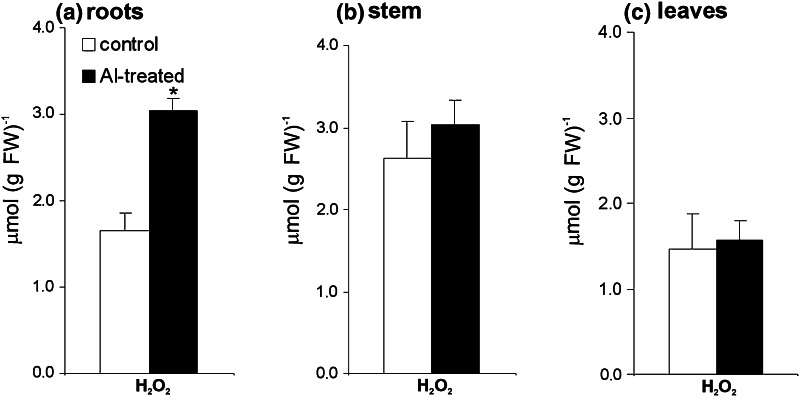
The effect of aluminum stress at 48 h, supplied as 250 μM AlCl_3_, on hydrogen peroxide accumulation in roots (**a**), stems (**b**) and leaves (**c**) of potato cv. ‘Bintje’. *Asterisks* indicate values that differ significantly from non-treated control potato plants at *P* < 0.05

The reduced NO formation in potato roots exposed to Al was accompanied by a statistically significant H_2_O_2_ accumulation (Fig. 4a). In turn, in the upper organs, i.e. stems and leaves, H_2_O_2_ remained at almost the same level as in the non-treated potato (Fig. 4b, c).

### The metabolic status of NO was affected during aluminum exposure

**Fig. 5:**
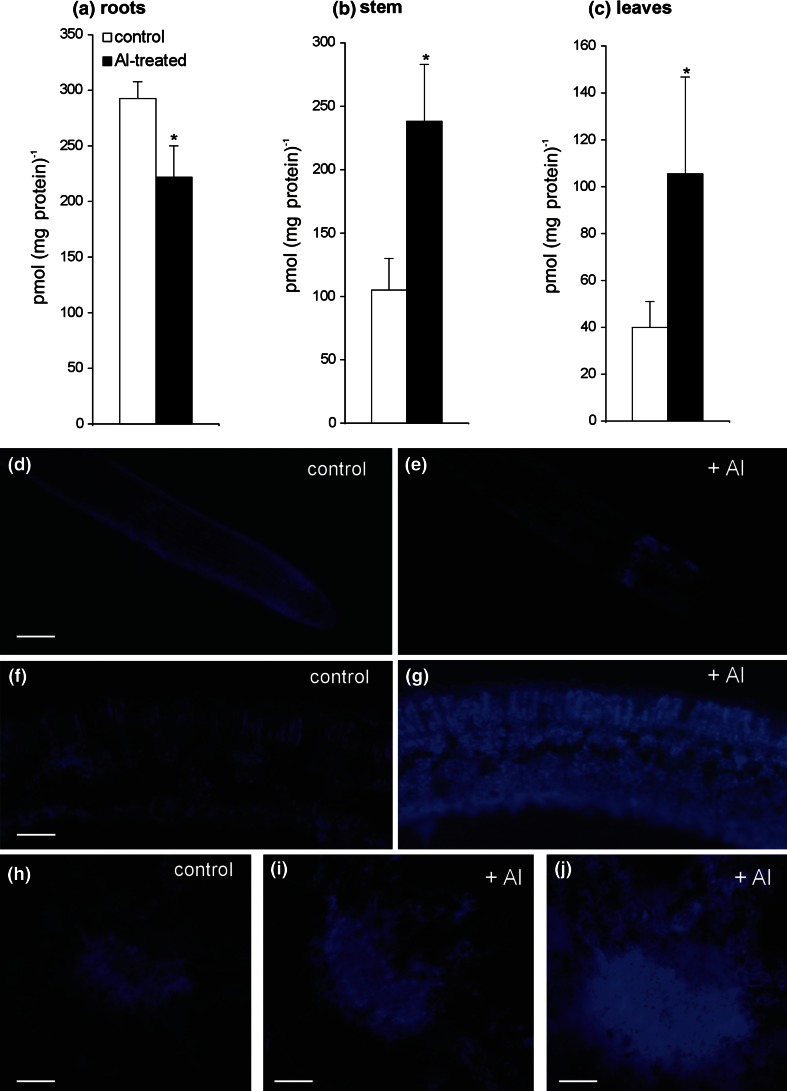
Total contents of *S*-nitrosothiols (SNOs) in roots (**a**), stems (**b**) and leaves (**c**) of potato cv. ‘Bintje’ treated with 250 μM AlCl_3_ or without AlCl_3_ (acidic control) at 48 h. Nitrosothiol content was determined by chemiluminescence using a Sievers^®^ Nitric Oxide Analyzer NOA 280i. Detection of SNOs in potato leaves by immunofluorescence histochemistry using Alexa Fluor 405 Hg-Link reagent phenylmercury. *Blue* fluorescence attributable to SNOs in roots (**d**, **e**) and leaves (**f**–**j**) of control and Al-treated potato. *Bars* indicate 250 μm (**d**, **e**), 200 μm (**h**, **i**), 100 μm (**f**, **g**, **j**). *Asterisks* indicate values that differ significantly from non-treated control potato plants at *P* < 0.05

To gain further insight into the participation of NO in the acquisition of the cross-resistance phenomenon between aluminum and biotic stress, the key parameters involving NO metabolism were analyzed. Based on a precise chemiluminescence method it was found that Al exposure reduced by approx. 25 % the total pool of SNOs in roots, whereas twofold higher SNOs production was observed in leaves and stems (Fig. 5a–c). Additional localization of SNOs using a fluorescent probe, Alexa Fluor 405 Hg-link, in control leaves showed blue fluorescence attributable to SNOs, present mainly in the phloem tissue (Fig. 5h). In leaves of Al-supplied plants the distribution of SNOs was expanded not only in the vascular tissue, but also in the spongy and palisade mesophyll (Fig. 5g, i, j). Moreover, the treatment with 250 μM AlCl_3_ for 48 h significantly diminished SNOs formation in potato roots (Fig. 5e). When *N*-ethyl maleimide (NEM) and Alexa Fluor 405 Hg-link were omitted in the incubation mixture, blue fluorescence was almost undetectable and similar results were obtained when the Alexa Fluor 405 Hg-link was added and NEM omitted (data not presented).

**Fig. 6:**
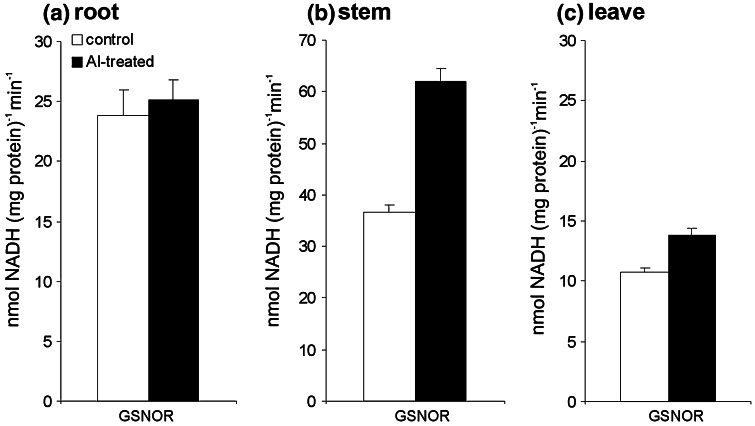
The effect of aluminum stress at 48 h, supplied as 250 μM AlCl_3_, on GSNO reductase activity in roots (**a**), stems (**b**) and leaves (**c**) of potato cv. ‘Bintje’. *Asterisks* indicate values that differ significantly from non-treated control potato plants at *P* < 0.05

The results suggest that cellular SNOs (mainly GSNO) homeostasis is controlled by GSNO reductase (Liu et al. 2001). In continuation of this experimental investigation our data showed that Al caused GSNOR activity up-regulation by approx. 20 and 45 % in leaves and stems, respectively (Fig. 6b, c). The enzyme activity in roots was comparable to the non-treated control (Fig. 6a).

### Aluminum exposure diminished late blight disease development on potato leaves

**Fig. 7:**
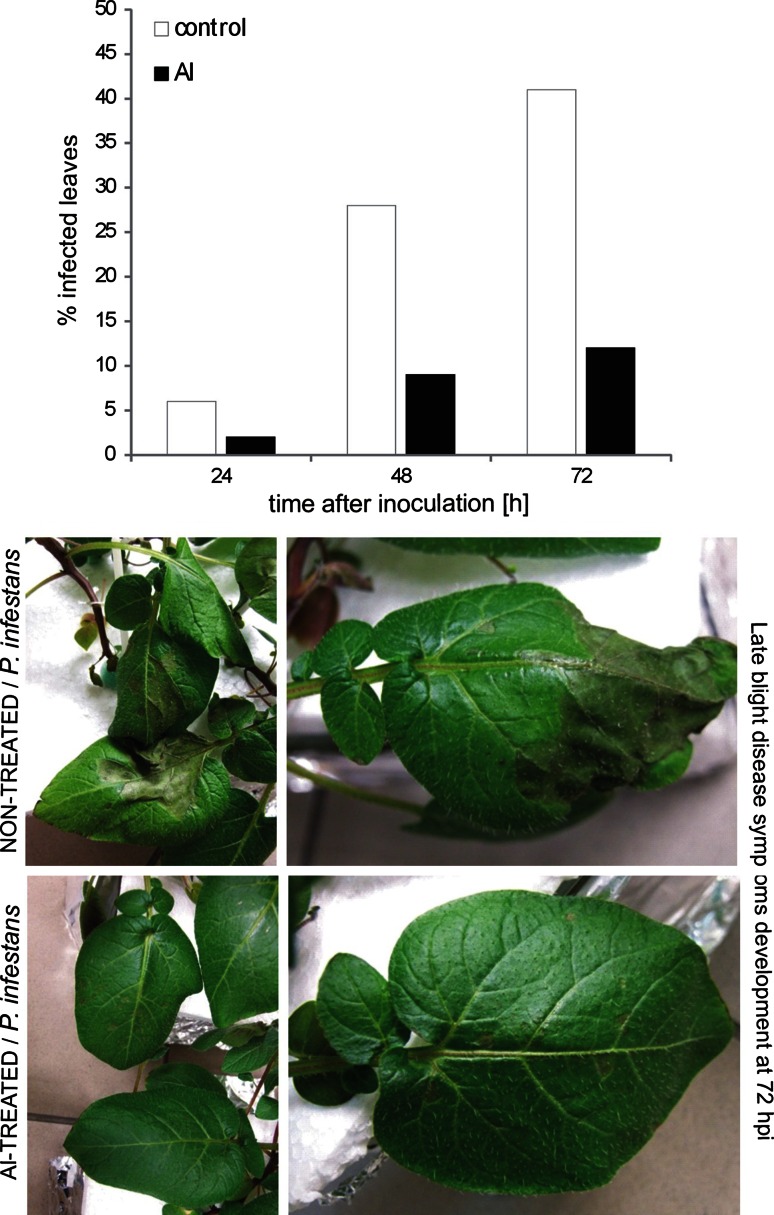
Systemic protection of potato cv. ‘Bintje’ against *P. infestans*. Potato plants were treated at root level with water (acid control) or exposed to aluminum stress for 48 h and then inoculated at the leaf level. The index of disease development in potato leaves at 24, 48, 72 h after *P. infestans* challenge inoculation (hpi) represents the percentage of leaf area covered by late blight symptoms. Values are means of the disease index of 20 leaves from three independent experiments

To assess the existence of a cross-resistance phenomenon between Al and *P. infestans*, potato plants were pretreated for 48 h with the metal at the root level and next inoculated with the oomycete pathogen at the leaf level. Based on the index of disease assay it was found that the metal effectively reduced symptoms of late blight disease. Pre-exposure to Al and a subsequent challenge inoculation showed disease limitation, resulting in approx. 20–30 % reduction of disease symptoms at 2nd and 3rd day after *P. infestans* treatment, respectively (Fig. 7).

### Aluminum enhanced molecular fingerprint accumulations of plant resistance in leaves after challenge inoculation with *P. infestans*

**Fig. 8:**
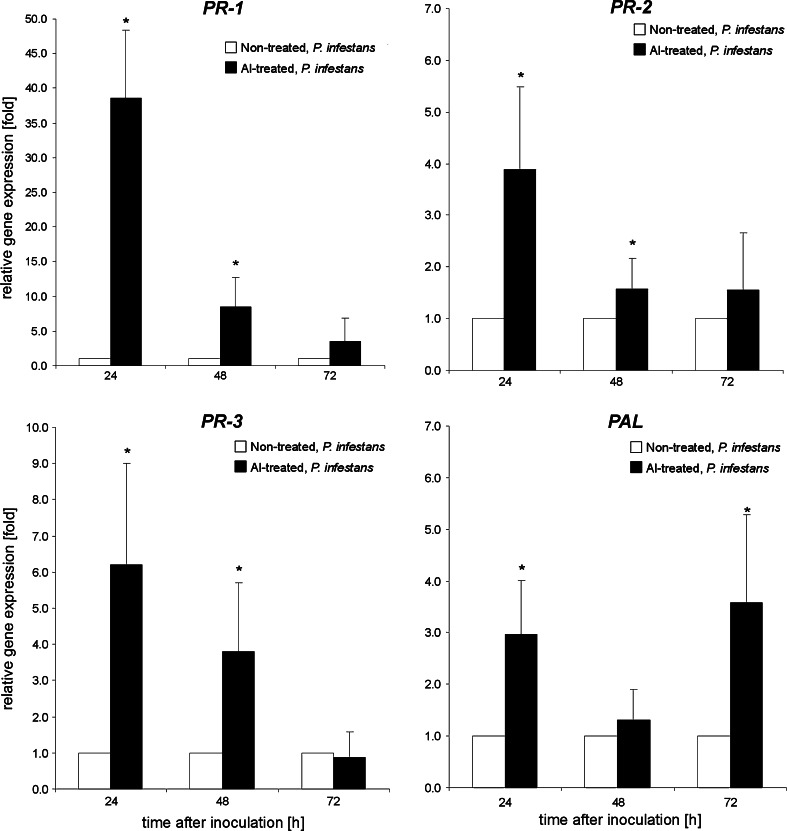
The effect of pretreatment with aluminum followed by challenge inoculation with *P. infestans* on *PR*-*1*, *PR*-*2*, *PR*-*3* and *PAL* gene expression in potato leaves. The qRT-PCR analyses of *PRs* and *PAL* were performed at 24, 48 and 72 h after challenge inoculation. *Asterisks* indicate values that differ significantly from non-treated, *P. infestans* inoculated leaves at *P* < 0.05, *n* = 3

The reduced disease symptoms observed after inoculation with *P. infestans* were correlated with an enhanced defense response in leaves. To highlight the effect of Al pretreatment during pathogen infestation we used non-treated (acid control), *P. infestans* inoculated leaves as a control to Al-treated, *P. infestans* inoculated leaves. The quantitative RT-PCR data revealed that Al-exposed, subsequently challenge-inoculated potato leaves showed a strong induction of genes encoding *PRs* and *PAL*. The expression of all studied defense-related genes was effectively up-regulated in response to pathogen challenge starting from 24 hpi (Fig. 8). The highest amount of mRNA (over 35-fold increase) coding for *PRs* was noted for *PR*-*1*. At the late stage of disease development, i.e. at 72 hpi, an enhanced expression was still noted for *PAL* gene (Fig. 8). The use of Al-treated, mock inoculated leaves as a control was presented in Supporting Information Fig. S4.

## Discussion

A wealth of evidence demonstrates that a high concentration of aluminum disturbs crucial physiological processes in plants, resulting in a variety of toxicity symptoms ranging from root growth inhibition to leaf chlorosis and necrosis. In turn, a general feature of active defense responses to this metal in plants depending on the Al dose might be executed by the stimulation of intracellular signaling pathways, leading to the expression of target genes, resulting in de novo synthesis of specific proteins engaged in plant protective or destructive metabolic changes.

### Aluminum triggered key hallmarks of plant defense in potato

In the present study, a short-term Al treatment of potato plants activated key markers of plant defense against pathogens, including *PR*-*1* and *PAL* at the root level. This finding is in line with previous reports showing that a number of PR genes and proteins including PR-4 like protein, thaumatin-like proteins, β-1,3-glucanase and chitinase were up-regulated in plant tissues directly exposed to Al (Mao et al. 2004; Chandran et al. 2008). The expression of *PR* families upon Al supply shows similarities to another induced defense response link to oxidative stress and lipid peroxidation, which have been shown to up-regulate PR proteins (Hong and Hwang 2005). Furthermore, it is known that class 1 of PR proteins, observed by us in Al-treated potato roots, is engaged in defense with antimicrobial activity ubiquitously synthesized by host plants in response to pathogen infections (e.g. Van Loon and Van Strien 1999) as well as by developmental signals (Lotan et al. 1989).

It was found in our study that in contrast to potato roots Al was unable to induce the expression of the *PR*-*1* gene at the distal leaf level. Our data revealed that in potato systemic leaves only *PR*-*2* and *PR*-*3* were effectively expressed at both mRNA and enzyme activity levels in response to root uptake of Al. Interestingly, the *PR*-*2* gene was also inducible in systemic leaves of *Arabidopsis* plants exposed to excess light (Mullineaux et al. 2000).

It has been reported that a pepper gene, *CABPR1*, which encodes the basic PR-1 protein, is induced after abiotic treatment including ethephon, wounding (Sarowar et al. 2005) and copper (Chmielowska et al. 2010). Moreover, transgenic tobacco overexpressing the pepper gene CABPR1 was found to confer resistance not only to pathogens, but also enhanced tolerance to heavy metals such as mercury and cadmium (Sarowar et al. 2005). These transgenic lines exhibited a significant decline in total peroxidase activity, suggesting that overexpression of CABPR1 in tobacco cells altered the redox balance, triggering an H_2_O_2_ dependent stress tolerance cascade of metabolic changes (Sarowar et al. 2005). Although the precise role of PR proteins in combating Al stress is far from being resolved, the sequence of events involving local changes in the redox balance might operate also in Al-exposed potato roots.

### Aluminum treatment induced local H_2_O_2_ accumulation

Root uptake of Al affected only local accumulation of H_2_O_2_. It is known that root cells as the first line controlling environmental factors might sense and then transfer the signals into the cell interior to trigger response in the whole plant. A local H_2_O_2_ over-accumulation observed in potato roots exposed to moderate and short-term Al stress was tuned with a diminished NO generation. The observed changes in the redox state in potato roots might create a cellular milieu facilitating distal signal generation.

Hydrogen peroxide senses stress signals and modulates the redox state, i.e. peroxidases, malate dehydrogenase, thiol redox-associated proteins, chitinase and PR-1a (Zhou et al. 2011). Zhou et al. (2011) identified 54 proteins regulated by H_2_O_2_ in apoplasts of rice roots. Remarkably, many of them are involved in carbohydrate metabolism. Thus, down-regulation of proteins involved in carbohydrate metabolism in response to H_2_O_2_ might be engaged in rearrangement of cell wall constituents. In turn, the H_2_O_2_ mediated up-regulation of UDP-glucose pyrophosphorylase, pectinesterase and α-arabinofuranosidase probably contributes to strengthening of the cell wall by influencing polysaccharide turnover and increasing pectin demethylesterification.

Al toxicity as an important growth limiting factor of root elongation is correlated with ROS overproduction (Yamamoto et al. 2003). However, the Al-induced abundant H_2_O_2_ production in roots of both Al-sensitive and Al-tolerant species has suggested that Al-dependent H_2_O_2_ accumulation could play a crucial role in the initiation and regulation of defense responses in roots (Xu et al. 2012). Members of different families of transcription factors, e.g. NAC, ZAT, WRKY, DREB, bZIP and MYB acting downstream of H_2_O_2_, are engaged in plant defense (Petrov and Van Breusegem 2012). Finally, overproduction of ROS, including H_2_O_2_ provoked by Al, can also induce cell death, as it has been observed in the root tips (Panda et al. 2008).

### Aluminum provoked the SA-dependent systemic pathway from roots to leaves

It is generally accepted that the essential role of SA is to activate plant defense responses and plant protection from pathogen attack (Malamy and Klessig 1992). SA-mediated signal transduction seems to be implicated in plant response to Al as well (Yang et al. 2003; Liu et al. 2012). SA might mediate Al-induced oxidative stress in addition to the major role of SA in regulation of Al responsive citrate efflux in plant roots by modulating aconitase and/or citrate transporters (Yang et al. 2003; Liu et al. 2012). In this study, we demonstrated for the first time a systemic activation of Al stress responses, since a relatively short Al treatment strongly induced free SA and its conjugated form—SAG accumulation not directly in roots exposed to the metal, but in systemic leaves. Thus, both SA and SAG levels significantly decreased in potato roots with a concomitant enhancement of H_2_O_2_ accumulation. It is tempting to speculate that upon exposure of potato root to Al, SA might be bound to catalase, becoming a form of inactivation and it would lead to a local H_2_O_2_ accumulation that in turn could facilitate systemic signaling. Up to date, the relation between CAT activity and SA has been well defined only in biotic stress (van Breusegem et al. 2001). Moreover, H_2_O_2_ and SA-mediated defense signaling partly overlap, since the SA positive feedback loop is essential for amplifying distal signal for resistance or tolerance in the upper zone of the plant (Arasimowicz-Jelonek et al. 2012).

Aluminum modulated not only SA signaling in the upper organs of potato, but also post-stress NO generation. It was suggested earlier that NO might be engaged in root signaling in plants exposed to Al (Tian et al. 2007). A study on the internalization of aluminum into root cells revealed an inhibition of NO production in the distal portion of the transition zone in Arabidopsis roots (Illes et al. 2006). However, our new finding is that Al exposure provoked root-to-leaf NO signaling, since NO-FL fluorescence revealed a diminished NO synthesis in roots and stems with a concomitant enhancement of NO generation in the vascular and surrounding parenchymal cells of potato leaves. In this way Al-induced NO production occurring only in distal, untreated parts of the plant could be associated with an augmented capacity for the rapid activation of defense responses after a subsequent stress event, e.g. a pathogen attack. It was demonstrated earlier that NO formation is implicated in the mechanism of distal signaling (Gaupels et al. 2008). Piterková et al. (2009) presented evidence that the transmission of a systemic response throughout the tomato plant was related to an enhanced NO formation in distal, non-inoculated leaves. Moreover, production of NO involving the vascular tissue and neighboring cells was assigned to signaling during plant response to salinity, heavy metals and defense-related compounds (Requena et al. 2005; Valderrama et al. 2007; Gaupels et al. 2008; Arasimowicz-Jelonek et al. 2012).

### Impact of Al on NO mediated SNO formation in potato leaves

Considering an augmented NO production detected in potato leaves we next focused on the examination of NO might be converted into SNO storage. Our results revealed that an enhanced NO synthesis found in non-aluminum treated parts of potato was accompanied by a significant rise in the SNO pool, which was evidenced by both chemiluminescence and immunohistochemical methods. SNOs are formed by the covalent attachment of NO to a protein and non-protein thiols. The S-nitrosylation reaction is a rapid and reversible redox-based protein modification, facilitating a rapid transmission of the NO message into a physiological response. Moreover, changes in the cellular pool of SNOs may determine the fate of plant immunity (Yu et al. 2012). It was recently stated by us that potato leaves exposed to SAR inducers showed a rise in NO generation in cooperation with a reversible abundance of the SNO pool (Floryszak-Wieczorek et al. 2012). Based on BABA-induced events it was stated that coding the NO message in SNO storage at a relatively low threshold together with histone *H2B* up-regulation created a short-term imprint activation facilitating acquired resistance of potato to *P. infestans*.

To assess whether the SNO level differed in various potato organs we measured the concentration of SNOs at 24 h after Al root uptake. The SNO level was strikingly reduced in roots, while in contrast it increased in potato stems and systemic leaves when compared with the non-Al-treated potato plant. We need to stress here a relatively high level of SNOs in potato stems in view of the transport function of SNOs, or mainly GSNO as a potential systemic resistance signal moving through the vascular system. In confirmation, the immunohistochemical approach revealed an abundant GSNO accumulation in the main vein of potato systemic leaves after a previous exposure to Al root uptake.

Fifteen years ago, it was proposed for the first time that GSNO might serve as a long-distance phloem-mobile signal for SAR creation (Durner and Klessig 1999). The inability of Arabidopsis to accumulate SNOs correlated with the impairment of SAR establishment in the plants overexpressing reductase of GSNO, both in local and systemic leaves, was documented by Rusterucci et al. (2007). What is more, there is evidence that NO itself might act as a priming-active molecule to induce systemic acclimation against salt stress (Tanou et al. 2009).

To verify if GSNOR controls SNO levels in potato pretreated with Al we measured the activity of this enzyme regulated in the NO system by the breakdown of GSNO and *S*-nitrosylated proteins. Aluminum evoked an intensified NO generation in systemic potato leaves, linked to the abundant formation of SNOs and up-regulation of GSNOR activity. The level of GSNOR activity in systemic leaves did not closely interfere with the SNO level, but correlated with SAR establishment in Al-induced potato plants.

The emerging evidence suggests that cellular SNOs are precisely controlled by NO synthesis and GSNO turnover mediated mainly by GSNO reductase (Liu et al. 2001). Moreover, GSNOR appears to be a key regulator of systemic defense responses in both wounding and pathogenesis (Espunya et al. 2012). Previously Espunya et al. (2006) documented the localization of GSNOR in the phloem companion cells and xylem parenchyma in *Arabidopsis* plants and showed a more abundant GSNOR activity in the vascular system of plants overexpressing GSNOR both in local and systemic leaves.

However, there is no doubt that GSNO has an important effect for defense or acclimation response in plants, while there are disagreements how GSNOR-governs SNO concentrations, which are able to manipulate the acquisition of resistance. Analyses of GSNOR knock-out plants displayed elevated global SNOs levels and these plants were compromised in non-host and R-gene resistance (Feechan et al. 2005), furthermore were abolished for thermotolerance and failed to grow and develop (Lee et al. 2008).

Exploring the role of denitrosylation in plant resistance it should be noted that GSNOR is mainly involved in the breakdown of GSNO and currently information is also lacking on other cellular specific reductases or lyases acting on protein SNOs (Liu et al. 2001; Malik et al. 2011). Recently it was proposed that the pool of SNOs governed by AtGSNOR1 increases during the development of plant disease resistance, but beyond a certain threshold level of SNOs both SA synthesis, SABP3 function and NPR1 activity are diminished, which negatively regulates the establishment of plant immunity (Malik et al. 2011; Yu et al. 2012).

### Aluminum promoted protection to *P. infestans*

The present study provides new data regarding the effect of Al on plant organisms, since we found that plant root pre-exposure to aluminum diminished late blight disease symptoms on potato leaves. It needs to be emphasized here that it was correlated with a strong up-regulation of *PR*-*1* in leaves early (24 h) after the challenge inoculation of Al-pretreated plants. Simultaneously, the other analyzed defense genes, both *PR*-*2* and *PR*-*3*, were also expressed at a definitely higher level after pathogen inoculation in comparison with Al-non-treated plants. The markedly faster and stronger defense responses upon *P. infestans* challenge in Al-exposed potato suggests that aluminum induced changes in the short-term biochemical imprint in potato leaves involving reversible *S*-nitrosylated protein accumulation.

There is an increasing body of evidence that induced or primed plants display a unique state of an enhanced capacity to mobilize faster and more potent defense responses to a subsequent abiotic or biotic stress (Floryszak-Wieczorek et al. 2012). For example, β-aminobutyric acid (BABA) treatment, a non-protein amino acid with a high potential as a priming agent, leads to an induction of *PR* gene expression at a much lower level than the one noted upon future contact with a challenging pathogen (Slaughter et al. 2012). Although priming is well established in relation to biotic stress, the analogous phenomenon under abiotic stress conditions, leading to a hardening or acclimation process, is poorly characterized (Molassiotis et al. 2010). Ton et al. (2005) showed that treatment with BABA triggered tolerance to salt stress by an augmented expression of ABA-dependent defenses.

In our experimental approach previous aluminum stress triggered up-regulation of *PR*-*1* local in root and systemic in leaves. Only when the second biotic stress occurred we found an amplified defense response tuned with a 35-fold increase in *PR*-*1* expression when compared with non-treated inoculated plants.

**Fig. 9:**
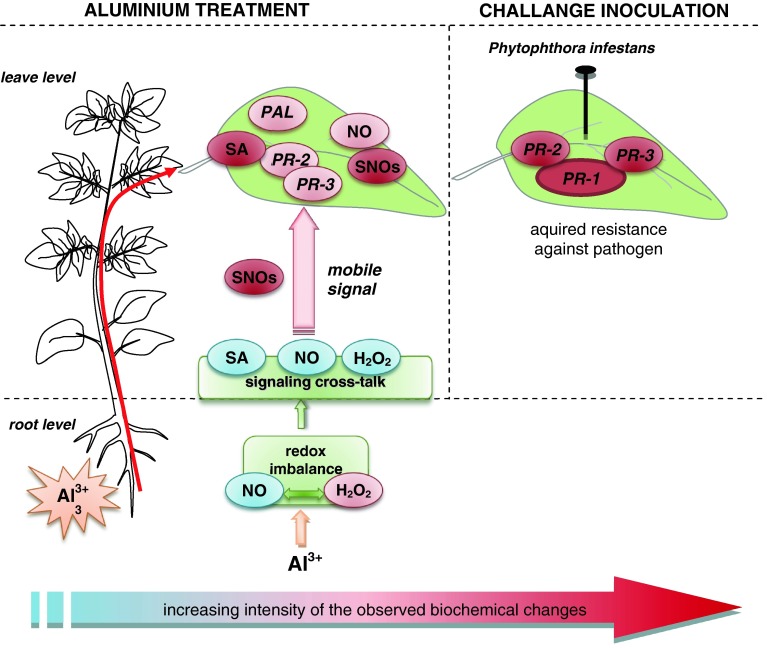
The proposed mechanism of Al induced cross-resistance of potato to *P. infestans,* in which moderate Al stress triggered changes in short-term biochemical imprint in distal potato leaves, facilitating effective defense responses against a subsequent pathogen attack. First, plant pretreatment with Al at the root level provoked redox imbalance manifested in H_2_O_2_ overproduction and diminished NO synthesis. These local changes might create a redox background for distal NO and SA-dependent signal generation. The complex regulatory networks facilitated systemic activation of Al stress responses engaged in the biochemical imprint linked to the coding of NO message in reversible SNO storage. Finally, signal amplification leading to potato resistance manifested in distal leaves of Al-treated plants was related to expression of SA-mediated defense genes (*PRs* and *PAL*) early after contact with a challenging pathogen and to subsequent disease limitation

Several lines of experimental data have shown that aluminum toxicity as the important growth limiting factor depends on its dose and is closely related to Al mobilization in soil promoted by low pH and acid rains. Our results revealed a new effect of Al potato root exposure on an enhanced resistance to a biotic factor (Fig. 9). It worth pointing that long-term responses are not directly caused by Al, but might rather be a consequence of numerous other Al-related biochemical and physiological processes, they might be more puzzling than short-term studies in determining the primary effect of Al. Based on this statement we evidenced that Al treatment directly conditioned the potato plant to express defenses rapidly upon *P. infestans* challenge.

## Electronic supplementary material

Below is the link to the electronic supplementary material.

**Supplementary material 1 (DOC 31** **kb)**

**Fig. S1** Relative growth rate of potato roots after 48-h incubation in various concentrations of AlCl_3_. For each treatment 30 plants were used in three independent experiments. Plants were incubated as described in Materials and Methods. **(JPEG 69** **kb)**

**Fig. S2** Bio-imaging of nitric oxide generation with Cu-FL fluorescent probe in potato plants treated with AlCl_3_ and AlCl_3_ + 1 mM PTIO, a specific NO scavenger. **(JPEG 216** **kb)**

**Fig. S3** Potato leaf cross section: palisade mesophyll (**1**), spongy mesophyll (**2**), parenchyma (**3**), xylem (**4**) and epidermal cells (**5**). Bars indicate 200 μm. **(JPEG 180** **kb)**

**Fig. S4** The effect of pretreatment with aluminum followed by challenge inoculation with *P. infestans* on PR-1, PR-2, PR-3 and PAL gene expression in potato leaves. The qRT-PCR analyses of PRs and PAL were performed at 24, 48 and 72 h after challenge inoculation. Asterisks indicate values that differ significantly from non-treated, mock inoculated leaves at *P* < 0.05 (*), *n* = 3. **(JPEG 367** **kb)**

## Abbreviations

Al: Aluminum
Cu-FL: Cupper-complex of 2-{2-chloro-6-hydroxy-5-[2-methylquinolin-8-ylamino)methyl]-3-oxo-3H-xanthen-9-l}benzoic acid
GSNOR: *S*-Nitrosoglutathione reductase
H_2_O_2_: Hydrogen peroxide
NO: Nitric oxide
SA: Salicylic acid
SAG: Salicylic acid beta-glucoside
SNOs: *S*-Nitrosothiols
