# Supplementary material for: Aluminum induces cross-resistance of potato to Phytophthora infestans
Source: Planta. 2013 Dec 18;239(3):679–94. doi: 10.1007/s00425-013-2008-8 (PMC3928512; doi:10.1007/s00425-013-2008-8)
Supplement: Supplementary file 1 — Supplementary material 1 (DOC 31 kb) [file 425_2013_2008_MOESM1_ESM.doc]

**Suppl. Table S1**: Primers used in real-time PCR reaction.

| Gene accession number in NCBI database | Coded protein | Primers |
| --- | --- | --- |
| AF043248.1 | PR-3  (chitinase) | Forward: ACTGGAGGATGGGCTTCAGCA  Reverse: TGGATGGGGCCTCGTCCGAA |
| AJ009932.1 | PR-2  (β-1,3-glucanase) | Forward: TTGGCCTTCTGAGGGACACCC  Reverse: GTGTTCCAGTCCCTCCTTTCACG |
| NM_001247429.1 | PR-1 | Forward: GAGCTGGGGACTGCAGGATGC  Reverse: CCGCGTTGAGCTGGGGGAAA |
| X63103 | PAL  (phenylalanine ammonia-lyase) | Forward: GTGCAAGAGCTGGTGGTGTGAAA  Reverse: AAGAGCACCACCATTTTTGG |
| X67238 | 18S RNA | Forward: GGGCATTCGTATTTCATAGTCAGAG  Reverse: CGGTTCTTGATTAATGAAAACATCCT |
| AB061263 | ef1α  (elongation factor) | Forward: ATTGGAAACGGATATGCTCCA  Reverse: TCCTTACCTGAACGCCTGTCA |
