# Supplementary figures and images for: Aluminum induces cross-resistance of potato to Phytophthora infestans
Source: Planta. 2013 Dec 18;239(3):679–94. doi: 10.1007/s00425-013-2008-8 (PMC3928512; doi:10.1007/s00425-013-2008-8)

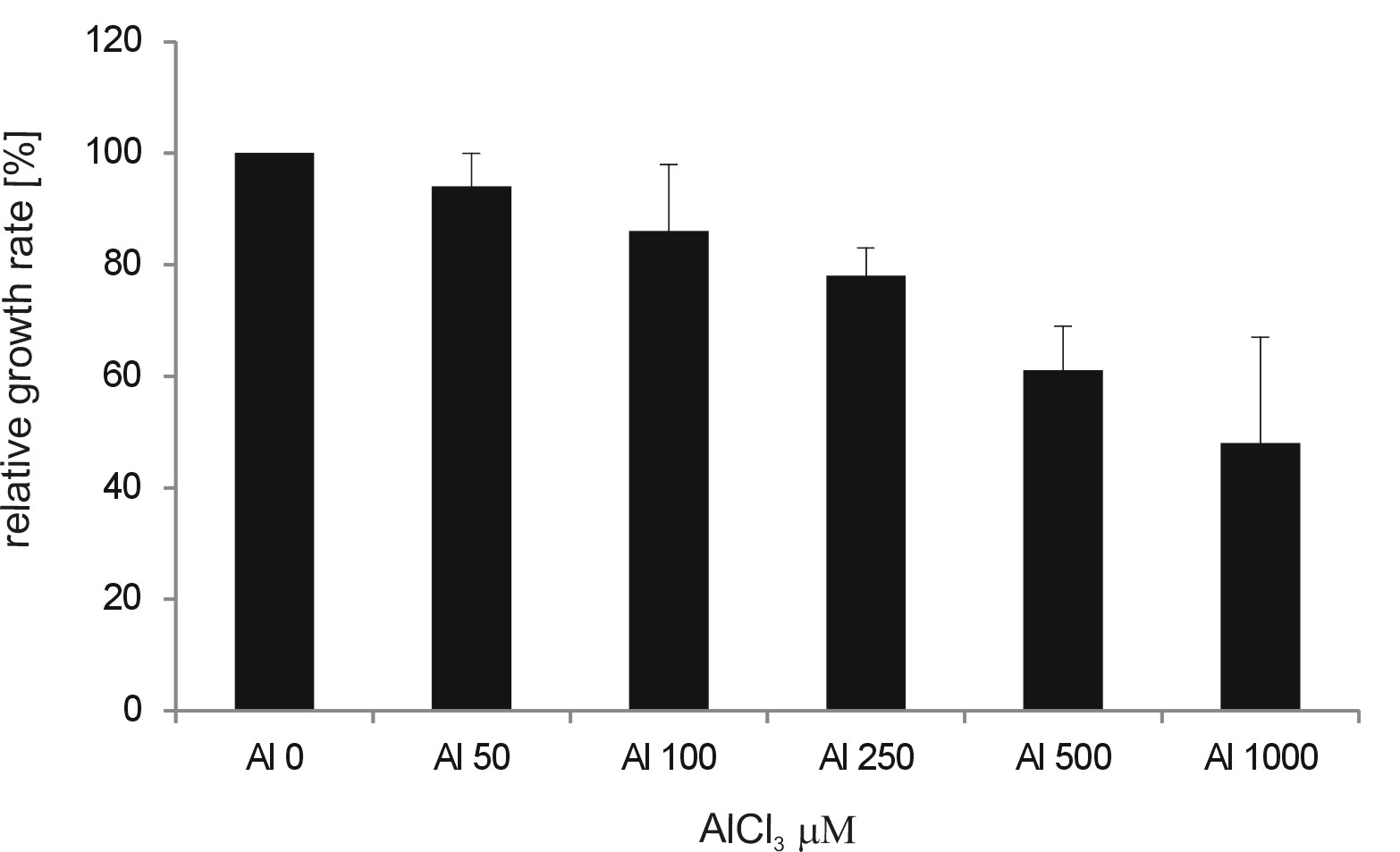

Supplement: Supplementary file 2 — Fig. S1 Relative growth rate of potato roots after 48-h incubation in various concentrations of AlCl3. For each treatment 30 plants were used in three independent experiments. Plants were incubated as described in Materials and Methods. (JPEG 69 kb) [file 425_2013_2008_MOESM2_ESM.jpg]

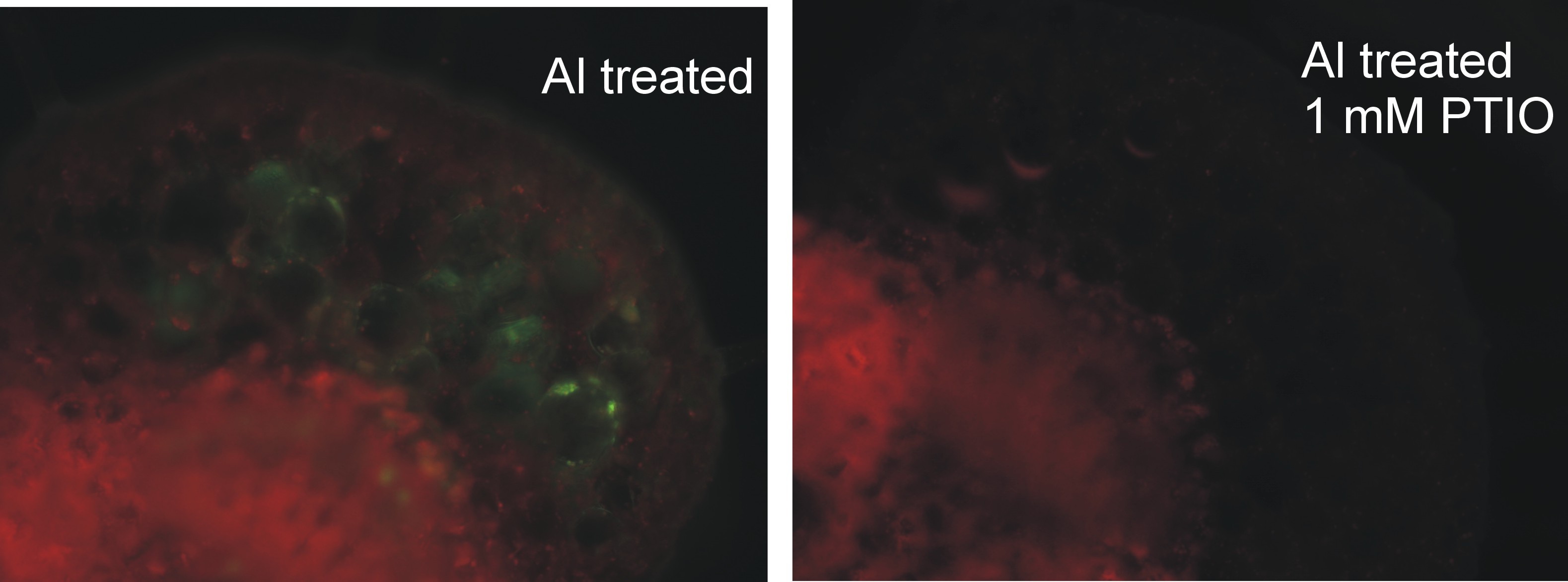

Supplement: Supplementary file 3 — Fig. S2 Bio-imaging of nitric oxide generation with Cu-FL fluorescent probe in potato plants treated with AlCl3 and AlCl3 + 1 mM PTIO, a specific NO scavenger. (JPEG 216 kb) [file 425_2013_2008_MOESM3_ESM.jpg]

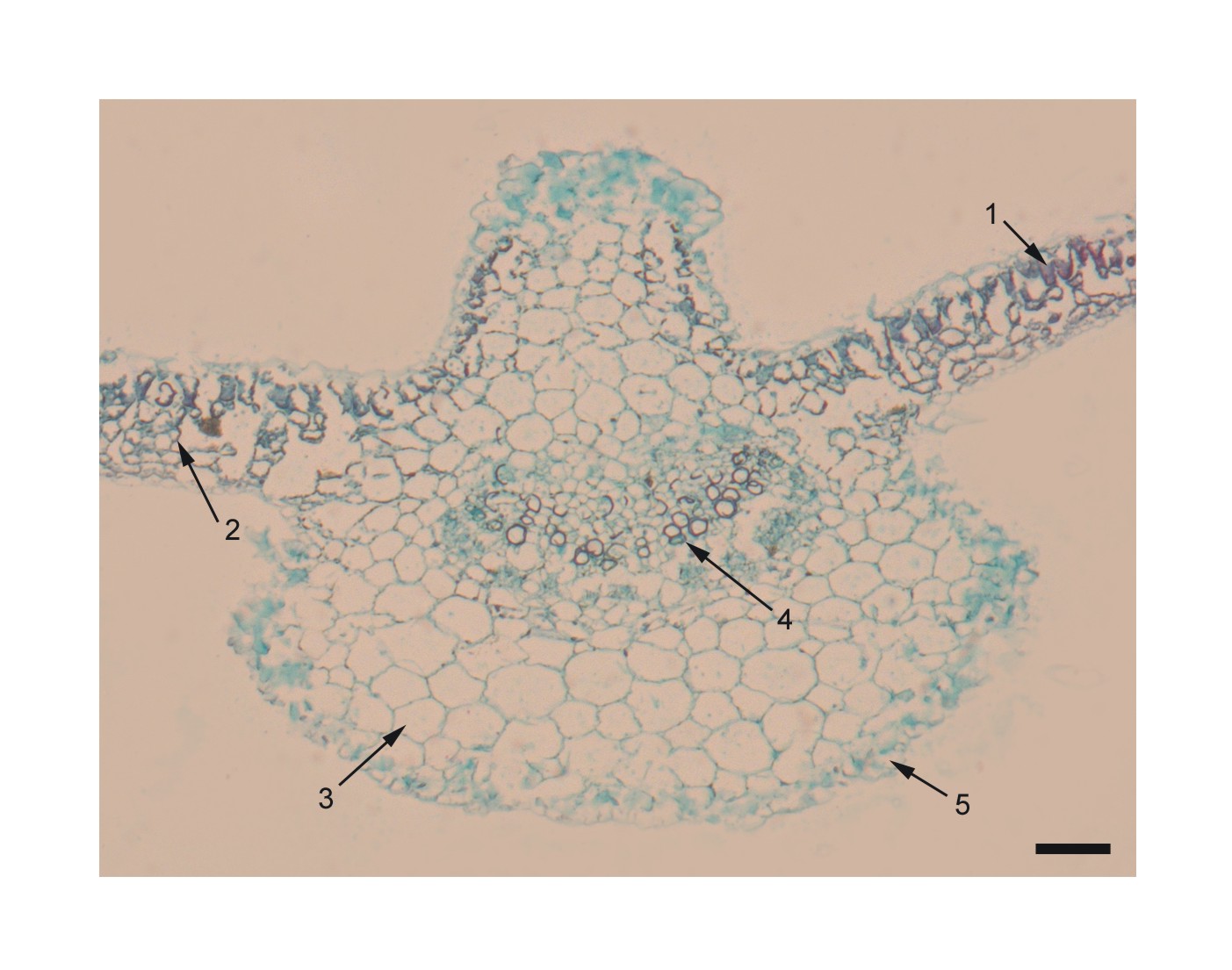

Supplement: Supplementary file 4 — Fig. S3 Potato leaf cross section: palisade mesophyll (1), spongy mesophyll (2), parenchyma (3), xylem (4) and epidermal cells (5). Bars indicate 200 μm. (JPEG 180 kb) [file 425_2013_2008_MOESM4_ESM.jpg]

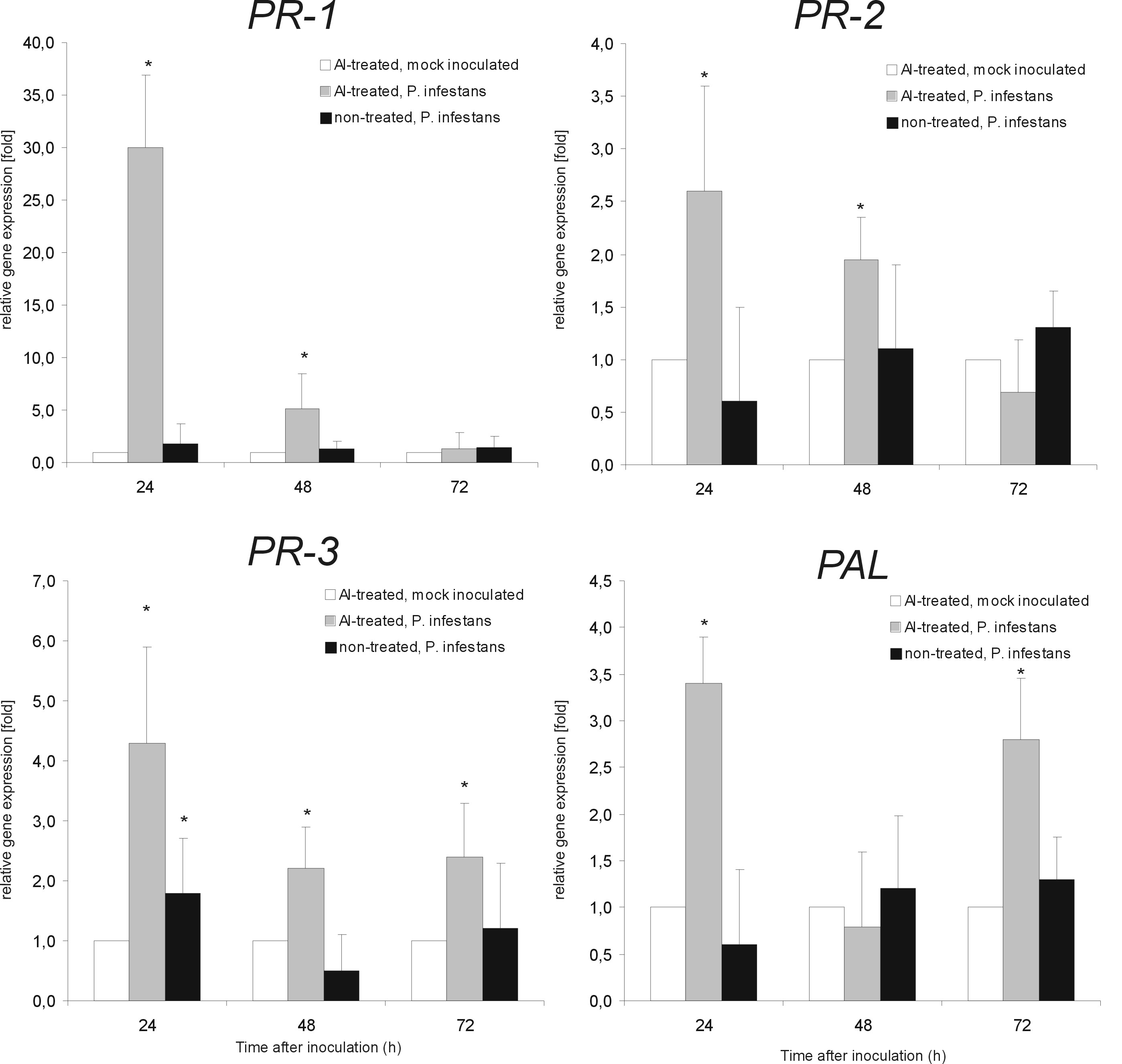

Supplement: Supplementary file 5 — Fig. S4 The effect of pretreatment with aluminum followed by challenge inoculation with P. infestans on PR-1, PR-2, PR-3 and PAL gene expression in potato leaves. The qRT-PCR analyses of PRs and PAL were performed at 24, 48 and 72 h after challenge inoculation. Asterisks indicate values that differ significantly from non-treated, mock inoculated leaves at P < 0.05 (*), n = 3. (JPEG 367 kb) [file 425_2013_2008_MOESM5_ESM.jpg]
